# QSAR Study for Carcinogenic Potency of Aromatic Amines Based on GEP and MLPs

**DOI:** 10.3390/ijerph13111141

**Published:** 2016-11-15

**Authors:** Fucheng Song, Anling Zhang, Hui Liang, Lianhua Cui, Wenlian Li, Hongzong Si, Yunbo Duan, Honglin Zhai

**Affiliations:** 1 Department of Public Health, Qingdao University Medical College, Qingdao 266071, China; qdsongfucheng@126.com (F.S.); qdlianghui@126.com (H.L.); qdlhcui@163.com (L.C.); lwenl27@163.com (W.L.); 2 Modern Educational Technology Center, Qingdao University, Qingdao 266071, China; anling_zhang@126.com; 3 Institute for Computational Science and Engineering, Laboratory of New Fibrous Materials and Modern Textile, The Growing Base for State Key Laboratory, Qingdao University, Ningxia Road 308, Qingdao 266071, China; bobduan@hotmail.com; 4 Department of Chemistry, Lanzhou University, Lanzhou 730000, China; zhaihl@163.com

**Keywords:** QSAR, aromatic amines, gene expression programming, multilayer perceptrons

## Abstract

A new analysis strategy was used to classify the carcinogenicity of aromatic amines. The physical-chemical parameters are closely related to the carcinogenicity of compounds. Quantitative structure activity relationship (QSAR) is a method of predicting the carcinogenicity of aromatic amine, which can reveal the relationship between carcinogenicity and physical-chemical parameters. This study accessed gene expression programming by APS software, the multilayer perceptrons by Weka software to predict the carcinogenicity of aromatic amines, respectively. All these methods relied on molecular descriptors calculated by CODESSA software and eight molecular descriptors were selected to build function equations. As a remarkable result, the accuracy of gene expression programming in training and test sets are 0.92 and 0.82, the accuracy of multilayer perceptrons in training and test sets are 0.84 and 0.74 respectively. The precision of the gene expression programming is obviously superior to multilayer perceptrons both in training set and test set. The QSAR application in the identification of carcinogenic compounds is a high efficiency method.

## 1. Introduction

Aromatic amines (AAs) are indispensable material in the process of synthesis azo colorants, which have strong tinting strength, bright color, and durability. So, the azo colorants have been widely applied to textile industry, food additives, cosmetics, and plastics [1,2]. In our life environment, we can come into contact with AAs at any time, such as gorgeous clothes, colorful food, and polluted air and water. The main ways of AAs to enter the body are the skins contact and digestive tract [3]. It is recognized that some AAs be verified or be suspected as human carcinogens. The enzyme P450 can help AAs convert into arylnitreniumlons in the body, which combine with C8 position of guanine in DAN.

Through extended exposure to the compounds, the structure of the DNA will be changed and a malignant tumor will appear. As a result, it leads to bladder, ureteral, renal, and pelvic carcinoma and other malignant diseases [4,5,6]. The European Commission Regulation 552/2009/EC has banned carcinogenic AAs to be used in textile and leather articles [7]. With the rapid development of chemical industry, a large number of compounds are produced and used. Compounds eventually are distributed in the environment by various uses, which strongly influence environmental and human health [8,9].

Due to the high carcinogenicity of AAs, recognition of the toxicity and carcinogenicity of the new AAs has special significance in toxicology. Thus, it is very important to assess the security risk of compounds. However, it is a huge project to assay the large number of compounds by experimental means. Toxicity identification of new compounds is very harmful to experimental animals. Even some experiments violate ethics requirements [10]. So, it is necessary to develop a simple, fast, and available approach to measure the property of security risk of compounds. The quantitative structure activity relationship (QSAR) method not only can quickly establish a reliable predicting model, but also can reveal the damaging effect mechanism of the poison interacting with the body and provide the reference information of designing and synthesizing safer and eco-friendly real green compounds [11]. Study [12] has been carried out with the aid of a combined quantum mechanics/molecular mechanics (QM/MM) computations to explore the detoxifying mechanism of agGSTe2 toward DDT. In this thesis, all AAs were randomly divided into a training set and test set, and then we set up prediction models based on molecular descriptors of AAs.

In the last two decades, many scholars had solved prediction problems by the establishment of bionic mathematics calculation model and the achievements were surprising [13]. Establishing a stability and rapid classification model is what we want. Gene expression programming (GEP) introduced by Ferreira [14] is an automatic programming approach, which overcomes certain limitations of genetic algorithms and genetic programming by working with two elements, the chromosome and the expression tree [15]. The advantage of GEP in designing decision trees makes it a successful method for solving classification problems [16,17]. Each physical and chemical parameter of AAs is as a gene unit in the gene expression programming. Complex algorithms weave them to a multivariate nonlinear equation. GEP in the field of carcinogenic classification shows incomparable superiority. The multilayer perceptrons (MLPs) is a biologically inspired computational tool for solving pattern recognition problems and is efficient in recognizing previously trained patterns. The capability of neural networks with multiple inputs and multiple outputs realizes data parallel processing and self-learning [13,18]. The parameters, as well as neurons, perform math functions intended to interweave them to a net, divided into carcinogens and non-carcinogens. In the current research, GEP and MLPs are new analysis strategies of the classification for carcinogenicity of AAs. Compared with MLPs, the proposed GEP is better in carcinogenic potency prediction of a suite of AA samples. 

## 2. Methodology

### 2.1. Source of AA Data

25 compounds have ionic pentavalent nitrogen atoms, and hexavalent sulfur atoms were eliminated, because the physical and chemical parameters cannot be computed, 128 fused ring aromatic amine (including heterocyclic compound) were taken from the literature [19], molecular structures and data of carcinogenicity are available. 1 stands for carcinogen, 0 stands for non-carcinogen. Carcinogenic activity is indicated by rat liver tumor. In this study, random allocation was taken to assure that every compound has the same opportunity to be divided into training set and test set. Each compound was given to a encoding, from 1 to 128. Then, 35 random numbers are generated in IBM SPSS 19.0 software (IBM Corporation, Chicago, IL, USA). If the encoding is same with the random number, this compound will be selected to test set. Finally, 128 compounds were divided into 93 training sets (Table 1) and 35 test sets (Table 2). The test set is used to evaluate stability of the QSAR model.

### 2.2. Calculation of Molecular Descriptors

In the QSAR model, molecular structure of compound was replaced by the corresponding physical and chemical parameters to establish numerical equations. All the structures of AAs were drawn into Chemdraw. Firstly, the geometry optimization operated in the Hyperchem 7.5 software (HyperCube Inc., Gainesville, FL, USA), the calculation used MM+ molecular mechanics force field. The semi-empirical AM1 method can get more precise optimization in the MOPAC. The molecular structures were optimized using the Polak-Ribiere algorithm until the root mean square gradient was 0.01 [20]. Then, the HIN files were generated by geometrical optimization, the MNO files were generated by MOPAC calculation. The CODESSA program (Semichem, Shawnee, KS, USA) can give the five classes of descriptors: constitutional, topological, geometrical, electrostatic, and quantum-chemical. Semi-empirical quantum chemistry methods are on the basis of the Hartree-Fock formalism, but include some approximations and obtain some parameters from empirical data. They are very appropriate for computational chemistry for treating physicochemical properties of large molecules. The semi-empirical AM1 calculation has shown to be successful in studying of QSAR. The constitutional descriptors show the molecular composition of the compounds without using the geometry or electronic structure; including number of atoms, molecular weight, and average atomic weight, etc. The topological descriptors are used to describe the atomic connectivity in the molecule; including wiener index, information content index and its derivatives, etc. The geometrical descriptors provide the information about the size of the molecule and require 3D-coordinates of the atoms in the given molecule; including shadow indices, molecular volume, etc. The electrostatic descriptors can reflect characteristics of the charge distribution of the molecule; including charged partial surface area descriptors, partial positive surface area, etc. The quantum-chemical descriptors will add important information to the conventional descriptors; including HOMO-LUMO energy gap, reactivity indices, etc. With the method of preprocessing, according to the following three rules [21], the model necessary descriptors are selected: (1) The parameters are the common of vast majority of the compounds; (2) For all the compounds, the descriptor numerical decreases; (3) The correlation coefficient of any two variables should <0.8. If any two descriptors have a correlation of 0.8, one should be removed. Otherwise, it will reduce the prediction efficiency of the QSAR model. This method could be used using well-established statistical projection technique such as PLS [22] or ACP [23] to construct uncorrelated variables.

### 2.3. Theory of Gene Expression Programming

Gene expression programming (GEP) is a new technique of novel algorithm for data mining that is based on the structure and function of biological gene [24]. It carries on all the advantages of both genetic algorithm (GA) and genetic programming (GP), by eliminating some of their own limitations. GEP adopts fixed length, nonlinear, or linear strings of chromosomes to solve complex problems by forming the expression trees of different shapes and sizes when evaluating their fitness [25]. The search space of GEP is separated from the solution space, which can be expanded to the benefits such as unconstrained search of the genome space, thus achieving the purpose of using simple coding to solve classification problems. 

**Figure 1:**
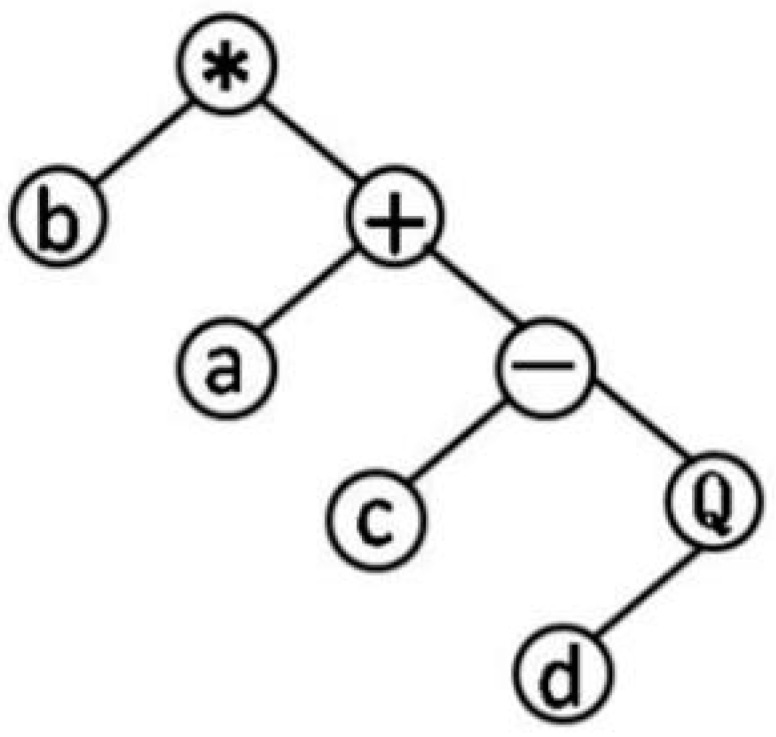
Expression trees.

GEP genotype individuals consist of the head and tail, the head elements from the function character and terminator sets, tail elements from terminator sets. The head is not strictly limited. The length of the head h is selected according to the number of parameters (such as a, b, c, 1, 2…) and the set of functions (such as sin, tan…). The common set of functions, F={+,−,∗,÷,Q}, Q represents the root function. The tail only contains the variable. The length of the tail t should be computed as: t=h(n−1)+1. *n* is the number of parameters for the maximum number variable function. The chromosomes function as a genome, after being modified by various means of mutation, transposition, root transposition, gene transposition, gene recombination, and one-point and two-point recombination, that will be transformed into expression trees. Figure 1 is one of the simplest expression trees can be processed into QSAR formula: $F=b*\left(a+\left(c-\sqrt{d}\right)\right)$. Parameters in the operation relationship were used to set up various models until get the best results. The application of complex functions can improve the prediction ability of QSAR model.

It is important that individuals to be selected and copied into the next generation according to the fitness function. The advantage of this kind of fitness function is the system that can find the optimal solution for itself. The calculation [26] for optimum fitness function (Equations (1)–(3)):

(1) $$fitness(R)=\left\{ \begin{array}{ll} 0, & \mathrm{if}\;consig\;(R)<0\\ consig\;(R)*\ln\;(compl\;(R)-1), & \mathrm{otherwise} \end{array}\right.$$

(2) $$consig\left(R\right)=\left(\frac{p}{p+n}-\frac{P}{P+N}\right)*\frac{P}{P+N}$$

(3) $$compl\;(R)=\frac{p}{P}$$

*p*, *n*, *P*, and *N* are number of all the positive compounds, number of all the negative compounds, number of the positive compounds in a training set, and number of the negative compounds in a training set, respectively.

For two classification prediction problems, only one GEP rule classification (*R*) can be achieved. Validating instances with GEP rules, if the result is positive, will be considered as a kind of the instance. Otherwise, it should be to the other kind. Exact representation is as follows:

If GEP_Rule (X) > 0 Then X ∈ class A
ELSE X ∈ class B
X stands for properties of instance.

**Figure 2:**
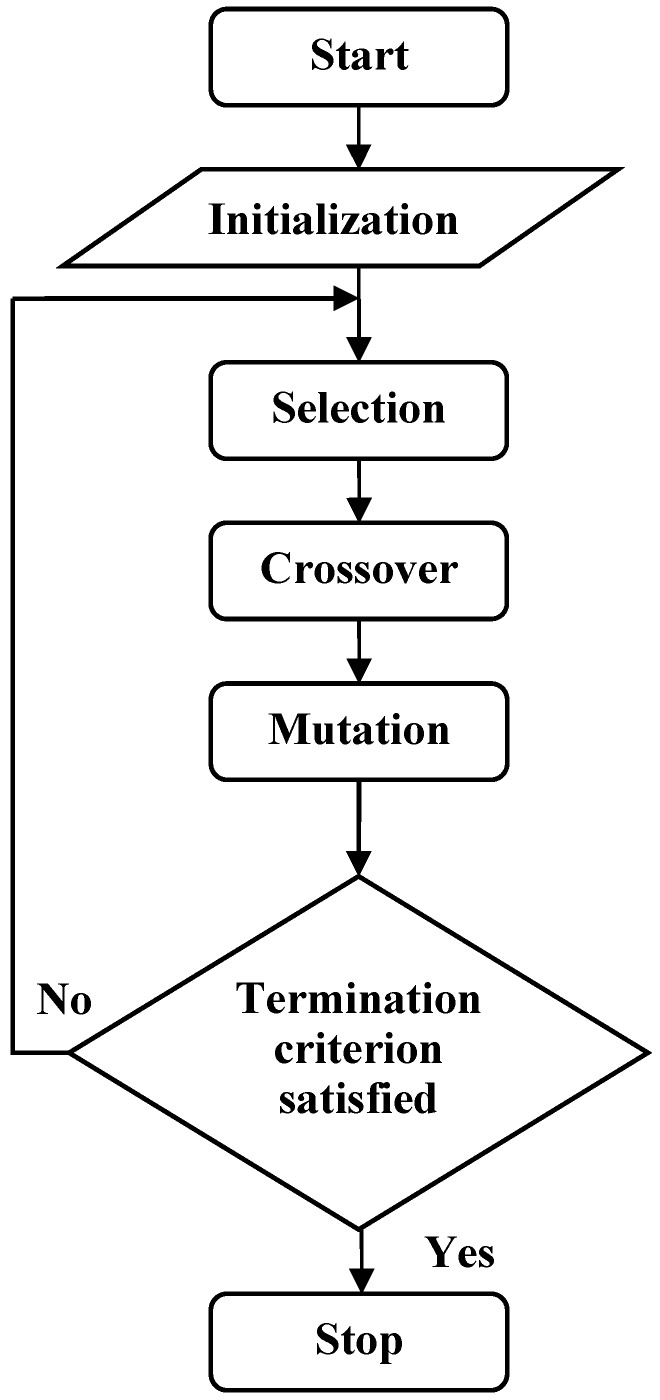
The flow chart of GEP.

The process of classification prediction problem is that decoding and calculating the fitness function of each chromosome, performing all kinds of genetic operation and updating chromosomes. This process will be repeated for a pre-established number of generations until the best model has been found [20]. Flow chart of GEP classification algorithm is shown in Figure 2.

### 2.4. Multilayer Perceptrons (MLPs)

**Figure 3:**
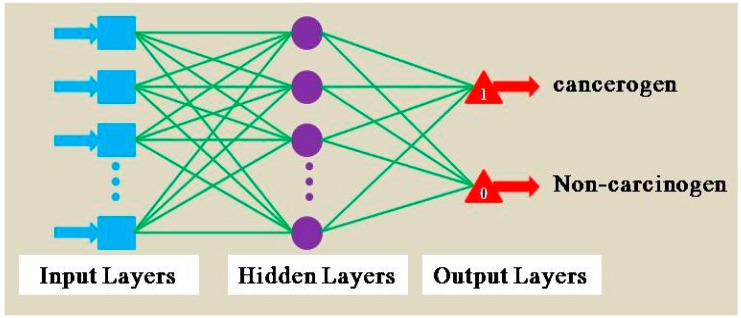
Multilayer perceptrons artificial neural network structure.

Artificial neural network (ANN) is based on the structure and function of neural network. It puts the complex neural network theory to simplify, abstract, and simulate. ANN has been widely used in classification, prediction, associative memory, pattern recognition, and other fields, which has gotten consistently high praise. What makes a MLPs different is that some neurons use a nonlinear activation function which is developed to model the frequency of action potentials, or firing, of biological neurons in the brain. Weka software provides a multilayer perceptrons artificial neural network. The use of back-propagation network algorithms makes MLPs application more expansive than other artificial neural networks. Figure 3 shows the structure of MLPs.

The input layer is decided by the dimensions of objects and the received signal is directly transmitted to the hidden layers. The number of hidden layers cannot be calculated by an accurate analytical formula and usually determined according to experience. In Weka, universal symbol “a” represents for hidden layers, $a=\frac{attribs+classes}{2}$. The realization of signal transmission and output of nodes between hidden layer and output layer is by excitation function [27]. Basic idea of carcinogenic classification forecasting of AAS by MLPs is that the known results of the sample model used for training network, and the carcinogenicity of compounds, can be identified by the trained network.

### 2.5. Platform of Weka

Waikato Environment for Knowledge Analysis (Weka) was developed by IanH. Wjttjn and EibeFrank of the University of Waikato and was based on JAVA software. As professional data mining software, Weka contains almost all of the classification methods in machine learning [28,29]. Under normal circumstances, the scholars are unable to preprocess the complex data without a good data analysis background of data mining. Weka provides a unified interface for users and saves manual programming for data analysis. Weka can not only provide a single classification algorithm of projections for the same data, but also can integrate several algorithms to predicting. To our knowledge, the rationale and complexity of classification algorithms can affect the accuracy of the prediction. Therefore, we chose different algorithm and combined the test of GEP and MLPs, so that we can obtain better prediction results and provide a good model. 

## 3. Results and Discussion

### 3.1. Significance of the Descriptors

Number of carbon atoms (NCOS): The number of benzene rings is associated with the carcinogenicity. Growing number of C atoms will increase the morbidity of cancer [30]. On the other hand, the binding of methyl with DNA can change the conformation of double helix and affect the transcription of protein, which then changes tumor suppressor genes and gene mutation increases the risk of cancer [31]. The number of C atoms in nitrobenzene as descriptors to build the QSAR model has important significance.

Number of nitrogen-atoms (NNOS): Aromatic amines metabolic activation sites on the amino N atoms. Preliminary metabolic activation occurs in the liver, including *N*-catalytic oxidation by cytochrome P450lA2 and *N*-acetylation by acetyl enzyme catalysis. This process produce *N*-hydroxy. The aryl amines generated from oxidation can form additions with DNA to the urinary tract epithelial cells. Likewise, the structure of DNA is changed. *N*-*O*-sulfate ester is formed after sulfur transfer with *N*-hydroxyl. Another way, the reaction of *N*-hydroxyl with acetyltransferase produces *N*-*O*-acetate ester. The unstable *N*-*O*-sulfate ester and *N*-*O*-acetate ester generate N ions in hydrolysis, which can combine with normal ion-making nucleophilic reaction with DNA bases [30,32]. Highly activated free radical nitrogen ions cause normal cell mutation.

Kier flexibility index (KFBI), Balaban index (BBI), structural information content index (order 0) (SICI), and topographic electronic index (all bonds) (TEIA) are topology descriptors. The molecular connectivity index as the structure characterization can provide a intuitive concept to make quantitative description on the molecular structure according to the molecular size, shape, and structure of chemical bond connection sequence and branched molecules—such as the structure of the information. The topology descriptors make structural differences quantitatively between the molecular quantitative and expression of molecular connectivity function. Different numerical topology values represent different molecular structures [32,33,34]. The four kinds of molecular descriptors are closely connected with carcinogenicity of AAs and can be used for the QSAR model. 

Polarity parameter (PLPT) is closely related to the solubility of molecules. The larger the lipo-hydro partition coefficient of low polar compounds, the higher the lipid solubility. It easily gets the lipid bilayer by simple diffusion and accumulates in adipose tissue. High polar compounds have better water-solubility. Water-solubility directly affects the toxicity and the target organ [35]. The polarity of aromatic amine determines the metabolism time in the body. The polarity parameters as discriminant factors are very crucial.

The lower the LUMO energy (LUMO) is more conducive to electrophilic reaction. Electrophilic reagents are related to the carcinogenicity of AA compounds. AAs could be converted to electrophilic reagents that are with some or all of the positive charge under the effect of cytochrome P-450 or other oxidase [36]. The atom with electrons in nucleophilic reagent easily reacts with the electrophilic reagents by sharing electrons. AAs as a promoter can enhance the carcinogenic effect of other poisons.

The correlation of eight descriptors is calculated by SPSS 20.0, in which any two variables related factor <0.8 (Table 3). It means that all variables are uncorrelated and not repetitive in the GEP models, so all the eight parameters could be adapted to QSAR study.

### 3.2. Results of GEP

128 compounds include 35 carcinogenic and 93 non carcinogenic. The number of carcinogenic and non-carcinogenic compounds is 24, with 64 in the training set, respectively. The setting of the function is {+,−,×,÷,Mod,Exp,Log,Sin,Tan}, eight groups descriptors were used to build GEP model in the Automatic Problem Solver 3.0 (Gepsoft Limited Company, Bristol, UK). It takes about 25 min to select a most optimal model. Prediction result of each compound, accuracy, positive predictive value and negative predictive value (Table 4) are given by APS. We converted the C++ function into Equation (4).

(4) $$\begin{array}{l} F\left(x\right)=x_{1}+\tan\left[\log\left(x_{8}+x_{5}\right)-\frac{x_{1}\cdot x_{6}}{x_{3}+x_{2}}\right]\\ +\frac{\tan\left(x_{5}+x_{3}\right)}{\mathrm{mod}\left[\log\left(x_{6}\cdot x_{2}\right),\log x_{8}\right]}+x_{5}+\\ \tan(x_{2}+x_{3}+x_{7}-x_{1})+\tan(x_{4}+x_{8}-x_{1})\\ +\tan\left\{\exp\left[\log(\frac{x_{5}}{\mathrm{mod}\left(x_{3},x_{1}\right)})+x_{5}\right]\right\} \end{array}$$

The variables $x_{1}$, $x_{2}$, $x_{3}$, $x_{4}$, $x_{5}$, $x_{6}$, $x_{7}$, and $x_{8}$ represents the Number of C atoms, Number of N atoms, Kier flexibility index, Balaban index, structural information content index (order 0), and topographic electronic index (all bonds), polarity parameter, and the lower LUMO energy.

This is a complex nonlinear function, but classification prediction result is pretty better. Accurate rates of training set and test set are 0.92 and 0.82.

### 3.3. The Results of MLPs

Hidden layers were set “a”, training time is 500, validation threshold is 20. The test set is same with that of GEP. The training set is used to adjust the parameters of the model, and the test set is used to evaluate the predictive power. MLPs use the back-propagation algorithm and keep regulating weights in training to get the global error minimized.

**Figure 4:**
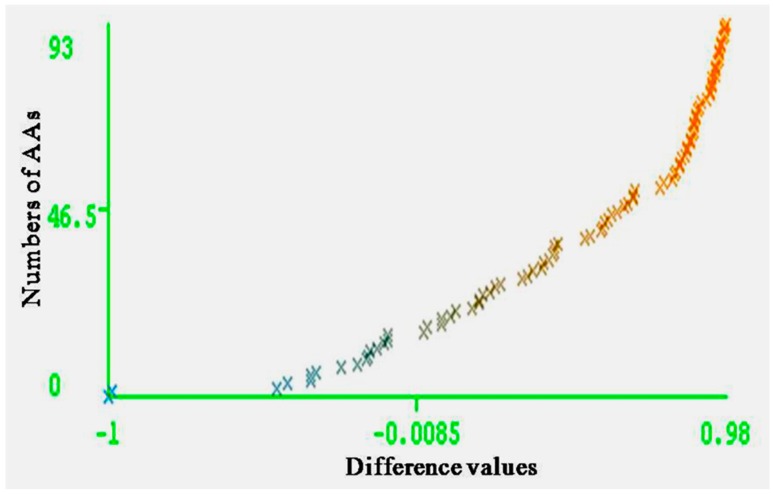
Curve margin of training set. The vertical axes represent the numbers of AAs; the horizontal axes represent the difference values of forecasting probability of actual categories, and the maximum prediction probability of wrong categories.

**Figure 5:**
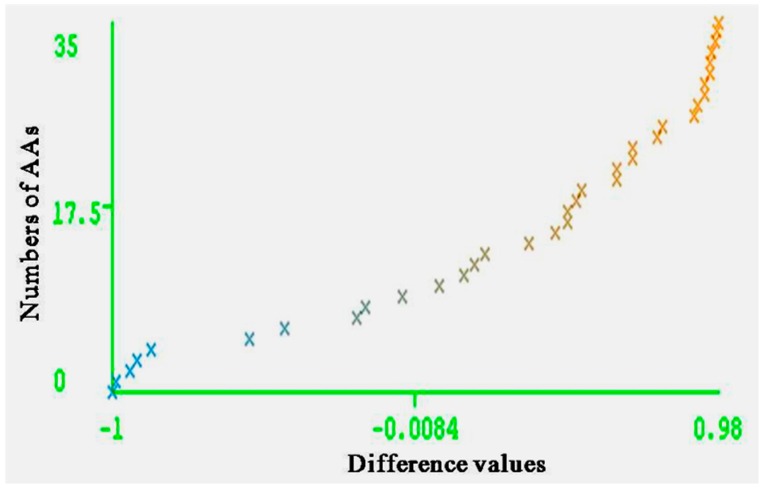
Curve margin of test set. The vertical axes represent the numbers of AAs; the horizontal axes represent the difference values of forecasting probability of actual categories and the maximum prediction probability of wrong categories.

The entire range of carcinogenic aromatic amine prediction accuracy is 0.84 of training set and 0.74 of test set by MLPs. Grid square represents error prediction. Curve margin could intuitively reflect the quality of classification prediction results. Curve margin is the difference values of forecasting, probability of actual categories, and the maximum prediction probability of wrong categories. The vertical axes represent the sequence numbers of AAs. The horizontal axes represent the difference values. The greater difference values of samples closer to 1, the better classification effect. Figure 4 and Figure 5 show the vast majority of marginal values are close to 1. These two pictures indicate MLPs can accurately predict the carcinogenicity of AAs. The results of MLPs are given by Weka (Table 4). From the point of view of running time, 0.08 s for training set and 0.20 s for the test set.

### 3.4. Comparison between GEP and MLPs

The purpose of this study is to establish a precise prediction model, to accurately identify the potential carcinogen of AAs. Carcinogenic compound prediction is very rare in previous studies. The GEP model based on human gene expression could accurately identify the carcinogenic of AAs. Performance assessment of classification algorithm shown in Table 4 uses recognized indicators precision, sensitivity, specificity, and Youden’s index obtained by optimizing Equations (5)–(7). These indexes are cited from “screening test” of epidemiology. Screening test has been widely employed in seeking potential patients to provide medical help in time. The indexes (accuracy, sensitivity, specificity, and Youden’s index) can show the reliability of screening tests. Our study combined QSAR and screening test methods from epidemiology.

(5) $$\mathrm{sensitivity}=\frac{A}{A+C}$$

(6) $$\mathrm{specificity}=\frac{D}{B+D}$$

(7) $${\mathrm{Youden}}^{\prime }s\;\mathrm{index}=(\mathrm{sensitivity}+\mathrm{specificity})-1$$

where A and B are the number of carcinogenic compounds predicted correctly and wrongly by QSAR model, C and D are the number of non-carcinogenic compounds predicted wrongly and correctly by QSAR model, respectively. All these indexes are cited from screening of epidemiology.

GEP is significantly better than MLPs. This is mainly because GEP algorithm could construct adaptive function by the evolution of its own and establish nonlinear relationship between the details and the carcinogenic compounds. Due to unique way of coding and genetic operation, GEP possesses remarkable ability to predict the carcinogenicity of AAs. So that the GEP algorithm will be more details to reflect differences in the resulting expression. GEP can give detailed predicted expressions while MLPs only provide prediction results. However, the GEP model is a complex nonlinear function and in the process of the establishment of the model is full of complications.

MLPs in the study results are not as good as GEP. To get a satisfactory result, GEP often taking a long time, but the MLP run time is within one second. For MLPs, there are no universal common rules specify how to set up training methods, build network structure and select the parameters. It adopted “trial and error” that large amounts of neural networks were tested until an optimal result was obtained. The network structure and parameter settings are usually through personal experience [37]. In addition, MLPs cannot accurately reflect the nonlinear relationship between multiple parameters. MLP are only used for existing AAs carcinogenicity data, but it cannot establish equation expressions to predict the properties of new compounds. The probability is obtained among the independence of each various property, but in practice this is not the case, it may lead to a decline precise rate. MLPs cannot give mathematical expression of the model. Although the computational time of GEP is much more than MLPs, the forecasting accuracy is more important within a certain range (computational time not too long).

## 4. Conclusions

The study on the carcinogenic compounds of compounds is essential in toxicology. The structure of the chemical compounds is the basis for the toxicity and effect the metabolism of toxic chemicals in the body. QSAR is an innovative idea to predict the carcinogenicity of AAs. QSAR can evaluate the superiority of the experimental group and give the valuable information for the risk assessment. In this study, the computational time of MLPs is lower than GEP, but the forecasting ability of GEP is better than MLPs. The unique advantage of GEP is that it can establish a mathematical model to predict the toxicity of new compound. In the design of AA compounds, it can increase or reduce the certain structure to achieve the purpose of reducing carcinogenic potential. Thus, GEP is a promising research direction in toxicology.

## Figures and Tables

**Table 1:** Carcinogenic activity of aromatic amines for training set.

No.	Aromatic Amines	Carcinogenicity (exp)	Carcinogenicity (GEP)	Carcinogenicity (MLPs)
1	*N*-Acetoxy-4-biphenylacetamide	0	0	0
2	*N*-Acetoxy-2-fluorenylacetamide	0	0	0
3	*N*-Acetoxy-4-phenanthrylacetamide	0	0	0
4	*N*-Acetoxy-*N*-(4-stilbenyl)acetamide	0	0	0
5	3-Amino-s-triazole	1	1	1
6	1-Anthramine	0	0	0
7	9-Anthramine	0	0	0
8	2-Anthranilacetamide	0	0	0
9	Benzidine	1	0	1
10	*N*-(Benzoyloxy)-fluorenylacetamide	0	1	0
11	4-Biphenyldimethylamine	0	0	0
12	3,6-Bis(dimethylamino)acridine	1	0	1
13	2-Chloro-4-phenylaniline	0	1	0
14	4′-Chloro-4-stilbenyl-*N*,*N*-dimethylamine	0	0	0
15	2-Cyano-4-stilbenamine	1	0	0
16	4,6-Diamino-2-(5-nitro-2-furyl)-s-triazine	0	1	1
17	0,0′-Dianisidine	0	0	0
18	3-Dibenzofuranylacetamide	0	0	0
19	3-Dibenzothiophenylacetamide	0	0	0
20	2,2′-Dichloro-4,4′-diaminostilbene	1	0	1
21	3,3′-Dichloro-4,4′-diaminostilbene	0	1	0
22	9,10-Dihydro-2-phenanthramine	0	0	0
23	3,3′-Dihydroxybenzidine	0	0	0
24	2-(4-(*N*,*N*-Dimethylamino)styryl) quinoline	0	0	0
25	3,2′-Dimethyl-4-biphenylamine	0	0	0
26	3,3′-Dimethyl-4-biphenylamine	0	0	0
27	2-Fluorenylacetamide	1	0	0
28	3-Fluorenylacetamide	0	0	0
29	1-Fluorenylaceto hydroxamic acid	0	0	0
30	2-Fluorenylaceto hydroxanic acid	1	0	0
31	*N*-Fluorenyl-2-benzamide	0	1	0
32	*N*-Fluorenyl-2-benzohydroxamic acid	0	0	0
33	2-Fluorenyldiacetamide	1	0	1
34	2-Fluorenyldimethylamine	1	1	1
35	2,5-Fluorenylenediacetamide	0	0	0
36	2-Fluorenylhydroxylamine	0	0	0
37	*N*-(2-Fluorenyl)-2,2,2-trifluoroacetamide	1	0	1
38	4′-Fluoro-4-biphenylamine	1	0	1
39	1-Fluoro-2-fluorenylacetamide	0	0	1
40	3-Fluoro-2-fluorenylacetamide	1	0	0
41	4-Fluoro-2-fluorenylacetamide	0	0	0
42	5-Fluoro-2-fluorenylacetamide	0	1	0
43	6-Fluoro-2-fluorenylacetamide	1	0	0
44	7-Fluoro-2-fluorenylacetamide	1	0	0
45	7-Fluoro-2-*N*-fluorenylacetohydroxamic acid	1	1	0
46	4′-Fluoro-*p*-phenylaniline	0	1	0
47	4′-Fluoro-4-stilbenamine	1	1	0
48	4′-Fluoro-4-stilbenyl-*N*,*N*-dimethylamine	1	0	0
49	2-Hydrazino-4-phenylthiazole	0	1	0
50	*N*-Hydroxy-*N*-(4-stilbenyl) acetamide	0	1	0
51	3-Iodo-2-fluorenylacetamide	0	0	0
52	7-Iodo-2-fluroenylacetamide	0	0	0
53	2-Methoxy-3-benzofuranylamine	0	0	0
54	7-Methoxy-2-fluorenylacetamide	1	0	1
55	1-Methoxy-2-fluorenylamine	1	0	1
56	3-Methoxy-2-fluorenylamine	0	1	0
57	4-((*p*-Methoxyphenyl)azo)-*o*-anisidine	1	0	1
58	2-Methyldiacetylbenzidine	0	0	1
59	4,4′-Methylenebis(2-chloroaniline)	1	1	1
60	4′-Methyl-4-phenylacetanilide	0	0	0
61	3-Methyl-4-phenylaniline	0	1	0
62	3-Methyl-4-stilbenamine	0	0	0
63	1-Naphthylacetohydroxamic acid	0	0	0
64	2-Naphthylhydroxylamine	0	0	0
65	9-Oxo-2-fluorenylacetamide	1	0	0
66	1-Phenanthrylacetamide	0	0	0
67	2-Phenanthrylacetamide	0	1	0
68	1-Phenanthrylamine	0	0	0
69	3-Phenanthrylamine	0	0	0
70	9-Phenanthrylamine	0	0	0
71	4-(Phenylazo) acetanilide	0	0	0
72	4-(Phenylazo) aniline	0	0	0
73	4-(Phenylazo) diacetanilide	0	0	0
74	4-(Phenylazo)-*N*-phenylacetohydroxamic acid	0	0	0
75	4-Stilbenamine	0	0	0
76	*N*-(4-Stilbenyl) acetamide	0	0	0
77	4-Stilbenyl-*N*,*N*-diethylamine	0	0	0
78	4-Stilbenyl-*N*,*N*-dimethylamine	0	0	0
79	*N*-(4-Styrylphenyl) hydroxylamine	0	0	0
80	3,2′,4′,6′-Tetramethyl-4-biphenylamine	1	0	0
81	*o*,*o*′-Tolidine	0	1	0
82	4-(*m*-Tolylazo) acetanilide	0	0	0
83	4-(*m*-Tolylazo) aniline	0	0	0
84	2-(*o*-Tolylazo)-*p*-toluidine	1	0	1
85	2-(*p*-Tolylazo)-*p*-toluidine	0	0	0
86	4-(*o*-Tolylazo)-*o*-toluidine	1	1	0
87	4-(*o*-Tolylazo)-*m*-toluidine	0	0	0
88	4-(*m*-Tolylazo)-*m*-toluidine	0	0	0
89	4-(*p*-Tolylazo)-*o*-toluidine	0	0	0
90	4-(*p*-Tolylazo)-*m*-toluidine	0	0	0
91	*N*,*N*,2′-Trimethyl-4-stilbenamine	0	0	0
92	*N*,*N*,3′-Trimethyl-4-stilbenamine	0	0	0
93	*N*,*N*,4′-Trimethyl-4-stilbenamine	0	0	0

**Table 2:** Carcinogenic activity of aromatic amines for test set.

No.	Aromatic Amines	Carcinogenicity (exp)	Carcinogenicity (GEP)	Carcinogenicity (MLPs)
1	2-Anthramine	0	0	0
2	4-Biphenylacetamide	0	0	0
3	4-Biphenylacetohydroxamic acid	0	1	0
4	3-Carbazolylacetamide	0	0	1
5	2,7-Diaminofluorene	0	0	1
6	4,4′-Diaminostilbene	1	1	0
7	2-Dibenzothiophenylacetamide	0	0	0
8	3,3′-Dichlorobenzidine	0	0	0
9	2-Fluorenamine	1	1	0
10	1-Fluorenylacetamide	0	0	0
11	3-Fluorenylaceto hydroxanic acid	0	0	0
12	2,7-Fluorenyldiacetamide	1	1	0
13	2-Fluorenyldiethylamine	0	0	0
14	*N*,2-Fluorenylformamide	0	1	0
15	2-Fluorenylmethylamine	1	0	0
16	*N*,2-Fluorenylsuccinamic acid	1	0	0
17	8-Fluoro-2-fluorenylacetamide	1	0	1
18	2-Fluoro-4-phenylaniline	0	0	0
19	3′-Fluoro-4-phenylaniline	0	0	0
20	3-Methoxy-4-biphenylamine	0	1	1
21	3-Methoxy-2-fluorenylacetamide	0	1	0
22	4,4′-Methylenebis(2-methylaniline)	1	0	1
23	3-Methyl-2-naphthylamine	0	0	0
24	2-Methyl-4-phenylaniline	0	0	0
25	2′-Methyl-4-phenylaniline	0	0	0
26	2-Methyl-4-stilbenamine	0	1	0
27	2-Naphthylamine	0	0	0
28	1-Naphthylhydroxylamine	0	0	0
29	9-Phenanthrylacetamide	0	0	0
30	2-Phenanthrylacetohydroxamic acid	0	0	0
31	2-Phenanthrylamine	0	1	0
32	4-(Phynylazo)-*o*-anisidine	1	1	0
33	1-(Phenylazo)-2-naphthylamine	0	0	0
34	4-(Phenylazo)-*N*-phenylhydroxylamine	0	0	0
35	3,2′,5′-Trimethyl-4-diphenylamine	1	0	1

**Table 3:** The correlation of eight descriptors.

Correlation	NCOS	NNOS	KFBI	BBI	SICI	TEIA	PLPT	LUMO
NCOS	1.000	−0.227	0.649	−0.708	0.667	0.234	−0.034	−0.374
NNOS		1.000	0.175	−0.014	0.159	0.312	−0.201	−0.111
KFBI			1.000	−0.569	0.730	0.433	0.007	−0.18
BBI				1.000	−0.681	−0.259	−0.173	0.438
SICI					1.000	0.620	0.250	−0.456
TEIA						1.000	0.339	−0.107
PLPT							1.000	−0.277
LUMO								1.000

**Table 4:** Results of GEP and MLPs.

	Accuracy	Sensitivity	Specificity	Youden’s Index
Training set of GEP	0.914	0.947	0.905	0.852
Test set of GEP	0.829	0.667	0.885	0.552
Training set of MLPS	0.838	0.844	0.813	0.657
Test set of MLPS	0.743	0.793	0.500	0.293
